# Tranexamic acid reduces heme cytotoxicity via the TLR4/TNF axis and ameliorates functional recovery after spinal cord injury

**DOI:** 10.1186/s12974-019-1536-y

**Published:** 2019-07-29

**Authors:** Shingo Yoshizaki, Ken Kijima, Masamitsu Hara, Takeyuki Saito, Tetsuya Tamaru, Masatake Tanaka, Dai-jiro Konno, Yasuharu Nakashima, Seiji Okada

**Affiliations:** 1 Department of Orthopedic Surgery, Graduate School of Medical Sciences, Kyushu University, 3-1-1 Maidashi, Higashi-ku, Fukuoka, 812-8582 Japan; 2 Department of Immunology and Neuroscience, Medical Institute of Bioregulation, Kyushu University, 3-1-1 Maidashi, Higashi-ku, Fukuoka, 812-8582 Japan

**Keywords:** Spinal cord injury, Tranexamic acid, Heme, Toll-like receptor 4, Demyelination, Hyperfibrinolysis

## Abstract

**Background:**

Spinal cord injury (SCI) is a catastrophic trauma accompanied by intralesional bleeding and neuroinflammation. Recently, there is increasing interest in tranexamic acid (TXA), an anti-fibrinolytic drug, which can reduce the bleeding volume after physical trauma. However, the efficacy of TXA on the pathology of SCI remains unknown.

**Methods:**

After producing a contusion SCI at the thoracic level of mice, TXA was intraperitoneally administered and the bleeding volume in the lesion area was quantified. Tissue damage was evaluated by immunohistochemical and gene expression analyses. Since heme is one of the degraded products of red blood cells (RBCs) and damage-associated molecular pattern molecules (DAMPs), we examined the influence of heme on the pathology of SCI. Functional recovery was assessed using the open field motor score, a foot print analysis, a grid walk test, and a novel kinematic analysis system. Statistical analyses were performed using Wilcoxon’s rank-sum test, Dunnett’s test, and an ANOVA with the Tukey-Kramer post-hoc test.

**Results:**

After SCI, the intralesional bleeding volume was correlated with the heme content and the demyelinated area at the lesion site, which were significantly reduced by the administration of TXA. In the injured spinal cord, toll-like receptor 4 (TLR4), which is a DAMP receptor, was predominantly expressed in microglial cells. Heme stimulation increased TLR4 and tumor necrosis factor (TNF) expression levels in primary microglial cells in a dose-dependent manner. Similarly to the in vitro experiments, the injection of non-lysed RBCs had little pathological influence on the spinal cord, whereas the injection of lysed RBCs or heme solution significantly upregulated the TLR4 and TNF expression in microglial cells. In TXA-treated SCI mice, the decreased expressions of TLR4 and TNF were observed at the lesion sites, accompanied by a significant reduction in the number of apoptotic cells and better functional recovery in comparison to saline-treated control mice.

**Conclusion:**

The administration of TXA ameliorated the intralesional cytotoxicity both by reducing the intralesional bleeding volume and preventing heme induction of the TLR4/TNF axis in the SCI lesion. Our findings suggest that TXA treatment may be a therapeutic option for acute-phase SCI.

**Electronic supplementary material:**

The online version of this article (10.1186/s12974-019-1536-y) contains supplementary material, which is available to authorized users.

## Background

Spinal cord injury (SCI) is a devastating neurological disorder that often leads to permanent functional and sensory impairments. In the pathology of SCI, primary mechanical trauma rapidly leads to the disruption of the blood-spinal cord barrier (BSCB) and results in intralesional bleeding as well as the infiltration of inflammatory cells [1]. To date, much attention has been focused on the role of various inflammatory reactions, and numerous experimental animal studies and clinical treatments using anti-inflammatory agents have been performed according to the pathology of SCI [2]. In contrast, the influence of intralesional bleeding on the pathology of SCI remains unclear.

Generally, immediately after physical trauma, the endothelial cells of injured vessel walls initiate a sequence of hemostatic reactions, including vessel contraction, platelet plug formation, coagulation cascade, and stable fibrin clot formation [3]. However after SCI trauma—despite the contribution of the hemostatic sequence—long-lasting substantial intralesional bleeding was observed for more than 7 days [4, 5]. This prolonged bleeding after SCI is partly attributed to local consumption coagulopathy and endogenous plasminogen activators similar to the hyperfibrinolytic-type disseminated intravascular coagulation (DIC) [6, 7]. Under this fibrinolysis-predominant condition, heme is released from the lysed red blood cells (RBCs) as it passes through the disrupted BSCB without the formation of stable fibrin clots [8]. Although heme plays an essential role in various biological processes as an active center of enzymes [9], heme is reported to exhibit cytotoxicity when it is released freely from hemeproteins, like hemoglobin in RBCs [10, 11]. Thus, in the present study, we assessed the cytotoxic effects of heme on the pathology of SCI.

Thus far, many therapeutic products have ever proposed to improve the life prognosis after hemorrhagic physical trauma—including, but not limited to, fresh frozen plasma, cryoprecipitate, platelet concentrate, fibrinogen, thrombin, prothrombin complex, recombinant factor VIIa, and so on. Among these, only tranexamic acid (TXA) is considered to be a reliable drug with evidence to support its reduction of the bleeding volume [12]. TXA can tip the balance of coagulo-fibrinolysis toward a coagulation predominant condition after the disruption of endothelial cells [13–15]. In fact, TXA is already available not only to reduce the perioperative bleeding risk [16–18] but also to reduce the post-traumatic bleeding volume [19]. We therefore hypothesized that TXA could also exert beneficial effects on lesional coagulopathies, such as hyper-fibrinolytic DIC after traumatic SCI.

In this study, we examined the effects of the administration of TXA in a mouse model of contusion SCI. Physiological and histological analyses revealed that heme released from the lysed RBCs was associated with the pathology of SCI. We also demonstrated that TXA could alleviate the intralesional bleeding and heme content, leading to the improvement of the pathology of SCI as well as locomotor functional recovery. Our findings suggest that the administration of TXA in the acute phase is a feasible therapeutic option for SCI.

## Methods

### Mice

Adult 8- to 10-week-old female C57BL/6 N mice (Japan SLC, Japan) were used in this study. All mice were housed in a temperature- and humidity-controlled environment on a 12-h light-dark cycle. All surgical procedures and experimental manipulations were approved by the Committee of Ethics on Animal Experimentation in the Faculty on Medicine, our University (A-28-256-0). Experiments were conducted under the control of the Guidelines for Animal Experimentation. All efforts were made to reduce the number of animals used in the experiments and to minimize their suffering. In this study, we used 92 mice in total.

### Spinal cord injury

Mice were anesthetized with pentobarbital (75 mg/kg, i.p.) and were subjected to a moderate (70 kdyn) contusion injury at the 9th thoracic level, which was made using an Infinite Horizons Impactor (Precision Systems Instrumentation, Lexington, KY), as previously described [2]. After injury, the overlying muscles were sutured, and the skin was closed with wound clips. During the recovery from anesthesia, the animals were placed in a temperature-controlled chamber until thermoregulation was re-established. Sham-operated controls were subjected to only laminectomy at the same level.

### Administration of tranexamic acid and heparin sodium in vivo

In TXA-treated mice, tranexamic acid (Transamin®, Daiichi Sankyo, Japan, 1 mg/g b.w., i.p.) dissolved in normal saline was administered immediately after spinal cord injury (SCI). In order to prevent the severe adverse effects of TXA, we diluted by 10 mg/ml and administered slowly at 1 ml/min. In heparin-treated mice, heparin sodium (Nacalai Tesque, Japan, 1 U/g b.w., s.c.) dissolved in normal saline was administered at 1 and 12 h after SCI. In saline-treated mice, normal saline was administered at 1 and 12 h after SCI. The schedule of the administrations is presented in an Additional file 1.

### Injection of autologous RBCs and heme into naïve spinal cords

Autologous blood (0.3 ml) was obtained from tail veins of naïve mice and washed three times in normal saline. The packed (non-lysed) RBC solution was prepared by centrifuging the autologous blood, discarding the supernatant including buffy coat, and diluting with normal saline to a total volume of 0.3 ml. To make the lysed RBC solution, the packed RBC solution was frozen in liquid nitrogen for 8 min, followed by thawing at 37 °C for 5 min three times, as previously described [20]. After laminectomy at the 9th thoracic level, the packed or lysed RBC solution was injected into naïve spinal cords at 0.5 mm lateral from dorsal veins using a 10 μl Hamilton syringe (Hamilton, Reno, NV) and a stereotaxic injector (KDS 310; Muromachi-kikai, Japan). The total injected volume was 2 μl at a rate of 1 μl/min. In order to prevent the flow back, the needle was kept in place for 2 min after injection. For the injection of heme solution, heme (hemin, ferric protoporphyrin IX, Nacalai Tesque, Japan) was appropriately diluted and was filtered at 0.22 μm to eliminate LPS contamination. Heme solution (125 or 250 μM) was also injected into the spinal cord at the 9th thoracic level, in the same way as the RBC solution was injected.

### Immunohistochemistry analyses

Mice were re-anesthetized and transcardially perfused with normal saline, followed by 4% paraformaldehyde (PFA) in 0.1 M PBS. The spinal cord was removed and immersed in the same fixative at 4 °C for 24 h. A spinal segment centered over the lesion epicenter was transferred into 10% sucrose in PBS for 24 h and 30% sucrose in PBS for 24 h and embedded in O.C.T. compound. The embedded tissue was immediately frozen in liquid nitrogen and stored at − 30 °C until use. Frozen sections were cut with a cryostat in the sagittal or axial plane at 16 μm, and mounted onto glass slides, as previously described [21]. On the other hand, the floating primary CD11b-positive cells were dehydrated on glass slides and fixed for 15 min in cold 4% PFA in PBS at room temperature and post-fixed in methanol at − 20 °C for 15 min. After washing three times with PBS, these sections and cells were used for the following immunofluorescent staining, as previously described [22]. For immunofluorescence staining, spinal cord sections and cultured cells were permeabilized with 0.01% Triton X-100 and 10% normal goat serum in PBS, pH 7.4, for 60 min. Then the sections were stained with primary antibodies against TER119 (1:200; red blood cell marker; rat, BioLegend), CD11b (1:200; microglia marker; rabbit, Serotec), NeuN (1:200; neuron marker; mouse, Chemicon), GFAP (1:200; astrocyte marker, rat, Invitrogen), GST-π (1:200; oligodendrocyte marker; mouse, BD Biosciences), and TLR4 (toll-like receptor 4; 1:200; rabbit, Abcam). The sections were then incubated with Alexa Fluor-conjugated secondary antibodies (1:200; Invitrogen). Nuclear counterstaining was performed using Hoechst 33342 (1:1000; Invitrogen). For luxol fast blue (LFB) staining, both an axial section around the lesion (from 2 mm rostral to 2 mm caudal) at 1 week after SCI, and a sagittal section at 4 days after the injection of RBCs or heme solution were prepared. These sections were stained, as previously described [23]. For counting apoptotic cells in each injured spinal cord, a terminal deoxynucleotidyl transferasemediated dUTP nick-end labeling (TUNEL) assay was performed using an ApopTag red in situ kit (Chemicon, Temecula, CA), as previously described [23].

### Quantitative analyses for immunohistology

For the quantification of the intralesional RBC volume, we performed a spectrophotometric assay using Drabkin’s reagent (Sigma-Aldrich, St Louis, MO, USA), as previously described [24]. Briefly, spinal cord tissue specimens (6 mm long) were obtained from freshly killed mice, and each spinal cord was treated individually according to the manufacturer’s instructions. Quantitative spectrophotometric hemoglobin assay was performed with Ensight (Perkinelmer Japan Co., Ltd). To prepare a cyanmethemoglobin standard absorbance curve, normal spinal cord tissue (6 mm long) was dissected from uninjured mice after blood removal by transcardially perfusion with normal saline and homogenized with Drabkin’s reagent containing incremental aliquots of blood. For quantification of the intralesional bleeding area, we obtained sagittal sections at 150 μm intervals from the injured spinal cord in each mouse and the TER119-positive area was measured using the ImageJ software program (National Institutes of Health). The area was calculated three times and the average value was taken, as previously described [21]. For the quantification of the LFB-positive area of the spared white matter, every 10th section was viewed on a light microscope (BZ-9000; Keyence, Japan) and the section containing the injury epicenter was defined visually as the one with the smallest visible rim of spared myelin. Then, LFB-stained serial sections from 2 mm rostral and caudal to the injury epicenter in each animal were imaged at × 40 magnification and captured. The LFB-positive area was determined as the percentage of pixels with the same grayscale value on the inverse image using the BZII-Analyzer measurement software program (Keyence). The LFB staining ratio of injured tissue compared with normal tissue was then determined, as previously described [25]. The ratio of CD11b/ TLR4 cells was calculated by counting the CD11b or TLR4-immunopositive cells in six sagittal sections at 140 μm intervals from 500 μm rostral and caudal to the injected site in each mouse (*n* = 6 in each group), using the Dynamic Cell Count BZHIC software program (Keyence). The number of apoptotic cells was also quantified by counting TUNEL-immunopositive cells in six sagittal sections at 140 μm intervals from injured spinal cord in each mouse (*n* = 6 in each group), using software Dynamic Cell Count BZHIC (Keyence). Heme content in the injured spinal cord and RBC solution were measured by using a modified QuantiChrom Heme Assay Kit (BioAssay Systems) according to the manufacturer’s instructions. The amount of heme in the lesion was expressed as μM per 500 μl lysate of 6 mm of spinal cord.

### Western blotting analyses

At 3 days after SCI, animals were re-anesthetized, and the spinal cords (4 mm centered around the lesion area) were rapidly removed and homogenized in lysis buffer (20 mM Tris-HCl [pH 8.0], 1 mM EDTA, 150 mM NaCl, 1% SDS, × 1/200 protease inhibitor cocktail [Nacalai Tesque], × 1/200 phosphatase inhibitor cocktail [Nacalai Tesque]). The supernatant was then dissolved in 4× Laemmli sample buffer, and sodium dodecyl sulfate-polyacrylamide gel electrophoresis was performed as previously described [25]. The membranes were then blocked for 1 h at room temperature with a blocking solution containing 5% BSA in TBS-T (20 mM Tris-HCl [pH 7.5], 150 mM NaCl, and 0.05% Tween-20) and incubated overnight at 4 °C with antibodies of TLR4 (1:2000; rabbit, Abcam), MyD88 (1:2000; rabbit, Abcam), NF-κB p65 (1:2000; rabbit, Santa Cruz Biotechnology), Hpx (1:2000; rabbit, Abcam), HO-1 (1:2000; rabbit, Assay Designs), Histone H3 (1:2000; rabbit, Cell Signaling), and β-actin (1:2000; mouse, Cell Signaling), diluted in a blocking solution. After washing with TBS-T, the membranes were incubated for 1 h at room temperature with horseradish peroxidase-conjugated anti-rabbit IgG (NA9340V; GE Healthcare, Bucks, UK). After washing with TBS-T, the membranes were reacted with Chemi-Lumi One Super (Nacalai Tesque) and detected by LAS-3000 (Fujifilm, Tokyo, Japan). The densitometric analysis of the Western blots (normalized to α-tubulin or Histone H3) was performed using the ImageJ software program (National Institutes of Health).

### Enzyme-linked immunosorbent assay

Segments of the spinal cord (4 mm) were rapidly dissected after circulating blood was removed. These spinal cord samples were then homogenized in sodium phosphate buffer (20 mM, pH 7.2) and protease inhibitor cocktail (Nacalai Tesque). After centrifugation at 15,000 rpm for 20 min at 4 °C, the remaining supernatants were used for measurements with a quantitative sandwich enzyme immunoassay technique. We measured the sample concentration of mouse TNF-α using an enzyme-linked immunosorbent assay (ELISA kit) (R&D systems) according to the manufacturer’s instructions with a multimode plate reader (Ensight; PerkinElmer Japan Co., Ltd., Chiba, Japan) at wavelength of 450 nm.

### Primary CD11b-positive cell culture and cell stimulation

Primary mixed glia cultures were prepared from pups on postnatal 3 day. Spinal cords were dissected and kept in ice-cold DMEM in 50 ml tube. Meninges, capillaries, and cerebellum were carefully removed, and the tissue was minced using a sterile scalpel. Digestion mix (7.5 ml [5 ml of 0.25% trypsin-EDTA; 2.5 ml of 1 mg/ml DNase I]) was added to the minced tissue (from six spinal cords) in 37 °C water bath for 10 min. FBS (2.5 ml) was added and centrifuged at 1000×*g* for 6 min; the pellet was re-suspended in complete media (DMEM; 10% FBS; 50 U/ml penicillin; 50 μg/ml streptomycin) and was filtered through 40 μm cell strainer. The cell suspension was seeded into poly-l-lysine-coated culture dish. Media was changed on in vitro day 5 to wash away unattached cells and media change was performed every 3 days thereafter, as previously described [26]. Magnetic sorting of CD11b-positive cells was performed using EasySep® Mouse CD11b-positive selection lit (STEM CELL Technologies, Canada) as per manufacturer’s instructions on in vitro day 14. The retained fractions were pooled and suspended in DMEM complete medium for downstream experiments. Cells were stimulated with 125 or 250 μM heme solution (Nacalai Tesque). After incubation for 30 min, cells were centrifuged at 1000×*g* for 5 min and the cell pellet was used for immunohistochemistry (IHC) analyses and mRNA extraction.

### Quantitative real-time PCR

We isolated total RNA from the injured spinal cord (6 mm long) and primary CD11b-positive cell culture (2 × 10^6^ cells/ml) using an RNeasy Micro Kit (Qiagen, Hilden Germany). RNA was primed with oligo dT primer and reverse transcribed using PrimeScript reverse transcriptase (TaKaRa, Japan) according to the manufacturer’s instructions. A real-time reverse transcription (RT)-PCR was performed using primers specific to the genes of interest (see Additional file 3) and SYBR Premix Ex Taqll (TaKaRa) in 20 μl of reactions. The levels of mRNA were normalized to the level of glyceraldehyde-3-phosphate dehydrogenase (GAPDH) mRNA for each sample, as previously described [22].

### Analyses of the locomotor function

First, the motor function of the paralyzed hindpaws was evaluated with a locomotor open field rating scale on the Basso Mouse Scale (BMS). Each mouse was assessed on 7 dpi and weekly thereafter until 6 weeks. A team of three independent examiners evaluated each animal for 4 min and assigned an operationally defined score for each hindpaw. Every test was performed in a double-blinded fashion, as previously described [23]. Second, for the foot print analysis, each mouse was determined to exhibit planter stepping or toe dragging at 6 weeks after injury, as previously described [21]. The forelimbs and hindpaws were dipped in red and green dyes, respectively. Then the animals were trained to walk on a paper-covered narrow runway (80 cm length and 4 cm width). A bright box was placed at the beginning of the runway and a dark box was also placed with their food at the end of track. Third, for the grid walk test, each mouse was evaluated using a 50-cm grid with three patterns: easy (50 steps, 1 cm apart), medium (every third step was removed), and hard (every other step was removed). The sum of the number of grips for all three patterns was used in the analysis, as previously described [23]. Finally, we further evaluated the locomotor function using a MotoRater® apparatus (TSE systems, GmbH, Germany). A mirror system allowed simultaneous locomotor quantification of different body sites by providing both the bottom and lateral views of the animals (Fig. 6d). Multi anatomic landmarks (iliac crest, hip, knee, ankle, and toe) were marked with small white beads (Fig. 6e) to facilitate a kinetic analysis using automatized TSE Motion High-Speed Video Analysis Software (Sophisticated Life Science Research instrumentation) [27]. In overground walking, the height of the iliac crest was defined as the vertical distance between floor and iliac crest marker on the lateral view (Fig. 6e). Stride width was defined as the distance from the left or right outermost hindpaw digit to the directional axis of overground walking (Fig. 6f). Paw rotation was defined as the angle between the axis of the rear paws and the directional axis (Fig. 6f). In swimming analyses, the water depth of swimming course was 25 cm and yellow floating rings were put on the tails of each mouse to increase their buoyancy (Fig. 6j). We traced the range of motion (ROM) with each landmark marker. The Iliac crest marker was made a reference point and we evaluated the relative positional relationship among the four markers. In addition to the toe ROM, we compared the ankle ROM among each of the mice. All measurements taken on each side for three consecutive steps were averaged. Every test was performed in a double-blind fashion.

### Statistical analyses

Statistical evaluations were performed with Wilcoxon’s rank-sum test. An ANOVA with the Tukey-Kramer post-hoc test was used for multiple comparisons between groups. *P* values of < 0.05 were considered to indicate statistical significance. In graphs, data are presented as the mean ± SEM. All statistical analyses were performed using the JMP software program (version 12; SAS Institute, Cary, NC, USA).

## Results

### The influence of the intralesional RBC volume on the SCI pathology

**Fig. 1:**
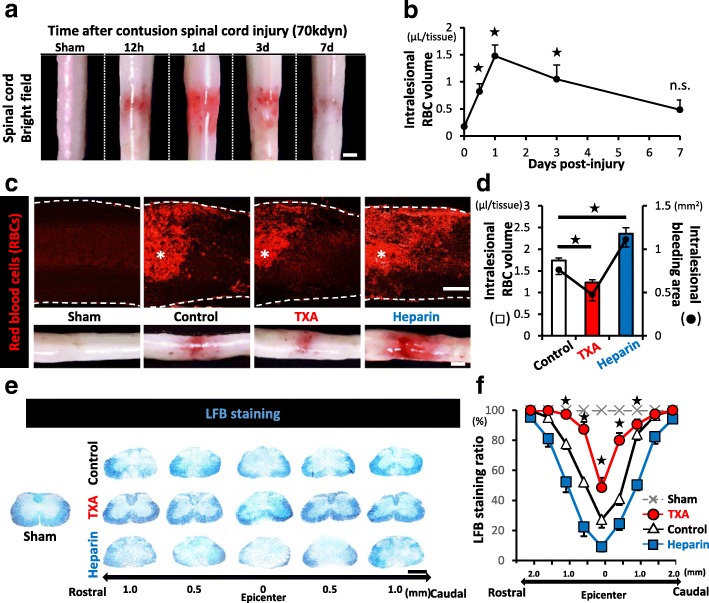
The intralesional bleeding volume is associated with the severity of demyelination after SCI. **a** The temporal macroscopic appearance of intralesional bleeding after SCI. **b** The time-course of the intralesional RBC volume after SCI (*n* = 4 at each time point). **c** IHC staining of sagittal sections of the injured spinal cord of sham-, saline-, TXA-, or heparin-treated mice at 1 dpi. * indicates the lesion epicenter. Intralesional RBCs are stained with TER119 (red). The insets at the bottom show representative bright field images of the each injured spinal cord. **d** The intralesional RBC volume (left *Y* axis: bar graph) and the intralesional hemorrhage area (right *Y* axis: dot graph) of the each injured spinal cord at 1 dpi (*n* = 8 in each group). **e** LFB staining of axial sections of each injured spinal cord from 1 mm rostral to 1 mm caudal to the lesion epicenter at 7 dpi. **f** LFB-positive spared myelin of (**e**) was quantified based on the LFB staining ratio (*n* = 6 in each group). Scale bars, 1 mm (**a**, **e**) and 500 μm (**c**).*p < 0.05, Dunnett’s test in comparison to sham mice (0 dpi) (**b**) and to saline-treated control mice (**d**), and one-way analysis of variance (ANOVA) with a Tukey-Kramer post-hoc test (**f**); n.s., not significant. Error bars indicate the SEM

To elucidate the influence of intralesional bleeding on the spinal cord injury (SCI) pathology, we first assessed the spatiotemporal change in the red blood cells (RBCs) in the lesions. Both macroscopically and quantitatively, we revealed that the intralesional RBC volume dramatically increased, reaching a peak at 1 day post-injury (dpi). Prolonged RBCs extravasation followed until the 7 dpi (Fig. 1a, b). Thus, we focused on 1 dpi as the most significant time point regarding the influence of intralesional bleeding in our model of contusion SCI.

We next investigated whether or not tranexamic acid (TXA) could reduce the intralesional bleeding and alter the following pathology. When we administered TXA immediately after SCI, the intralesional RBC volume and bleeding area at 1 dpi were significantly reduced in comparison to the saline-treated control mice (Fig. 1c, d). In contrast to the hemostatic drug TXA, we pharmacologically developed the hemorrhagic SCI mice by administration of heparin sodium (heparin), one of the popular anti-coagulant drugs. In the heparin-treated mice, the intralesional RBC volume and the bleeding area were significantly increased in comparison to those in the saline-treated control mice at 1 dpi (Fig. 1c, d).

Among these three groups (TXA-, heparin-, or saline-treated mice), we next compared the demyelinated area at 7 dpi using luxol fast blue (LFB) staining. In all groups, contusion SCI brought about demyelination, which was defined as the LFB-negative area in the dorsal parts around the lesion epicenter, and the demyelinated area in TXA-treated mice was significantly smaller than that in saline-treated control mice (Fig. 1e, f). In contrast, the demyelinated area in heparin-treated mice was significantly larger than that in saline-treated control mice (Fig. 1f). Since the severity of primary mechanical injury was equivalent among the three groups (see Additional file 2), these findings suggested that the demyelinated area was associated with the intralesional RBC volume and that the administration of TXA could ameliorate pathological conditions associated with SCI, such as demyelination.

### Hemolysis was involved in the SCI pathology

**Fig. 2:**
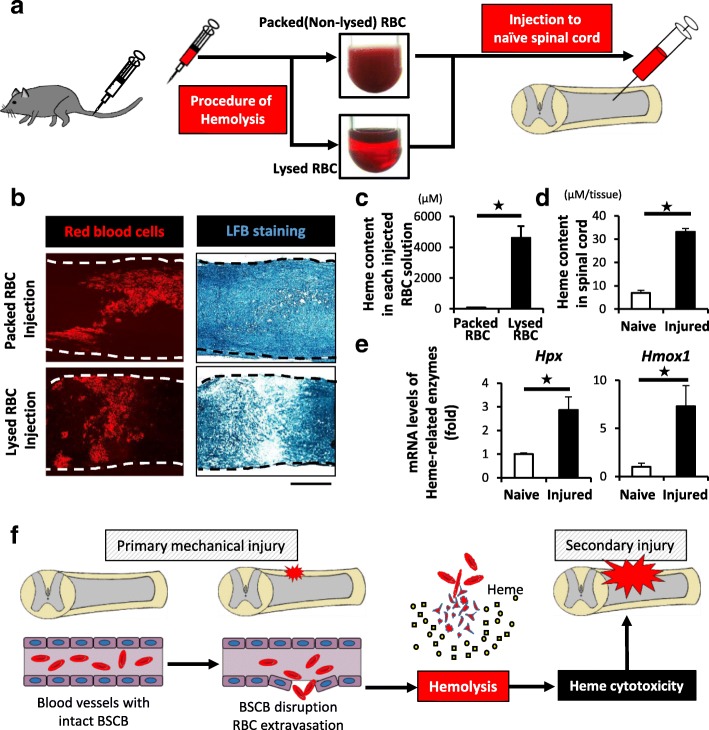
Intralesional heme released from lysed RBCs is involved in the pathology of SCI. **a** A schematic illustration of protocol of the autologous RBCs injection experiments. Packed (non-lysed) or lysed RBCs were injected into naïve spinal cords. **b** IHC and LFB staining revealed the TER119-positive RBCs (red) and LFB-negative demyelinated area on 4-day after the injection of lysed- or packed RBCs. Lysed RBCs were associated with demyelination around the injection sites. **c** The heme concentration of the lysed RBC solutions was significantly increased in comparison to the packed RBC solutions (*n* = 4 in each group). **d** The intralesional heme concentration in the injured spinal cords at 1 dpi was significantly increased in comparison to sham (laminectomy only) spinal cords (*n* = 6 in each group). **e** A qRT-PCR showed that the mRNA expression of Hpx and Hmox1 in the injured spinal cord was significantly upregulated in comparison to sham mice at 1 dpi (*n* = 4 in each group). **f** A schematic illustration of heme cytotoxicity after SCI. Scale bars, 500 μm (**b**). *p < 0.05, Wilcoxon rank-sum test (**c**–**e**). Error bars indicate the SEM

To examine the direct impact of intraparenchymal RBCs on the spinal cord, we injected the autologous RBC solution into naïve spinal cords and evaluated the demyelinating effect. Interestingly, there were little demyelination when the packed (non-lysed) RBC solution was injected; however, we found a prominent demyelinated area around the injected sites when the lysed RBC solution was injected (Fig. 2a, b).

Regarding the reason why only the lysed RBC solution had a toxic influence on the spinal cord, Dutra et al. reported that one of the toxicities of hemolyzed RBCs was attributed to heme, an iron complex released from hemoglobin in the pathological process of hemolytic diseases such as hemolytic anemia and sickle cell disease [9–11, 28]. Indeed, the heme content in the lysed RBC solution was much greater than that in the packed RBC solution (Fig. 2c). These results suggested that rather than the RBCs themselves, it was the heme from the hemolyzed RBCs in the lesions that was associated with the pathological process after SCI (Fig. 1e, f).

In fact, we confirmed that the intralesional heme content was significantly increased in the lesion area after SCI (Fig. 2d). In addition, we revealed that the expression levels of heme-related enzymes, hemopexin (Hpx) and heme oxygenage-1 (Hmox1), were also significantly increased at the lesion area (Fig. 2e), indicating that intralesional hemolysis was actually involved in the SCI pathology (Fig. 2f).

### Heme upregulated the TLR4 expression in CD11b-positive microglial cells

**Fig. 3:**
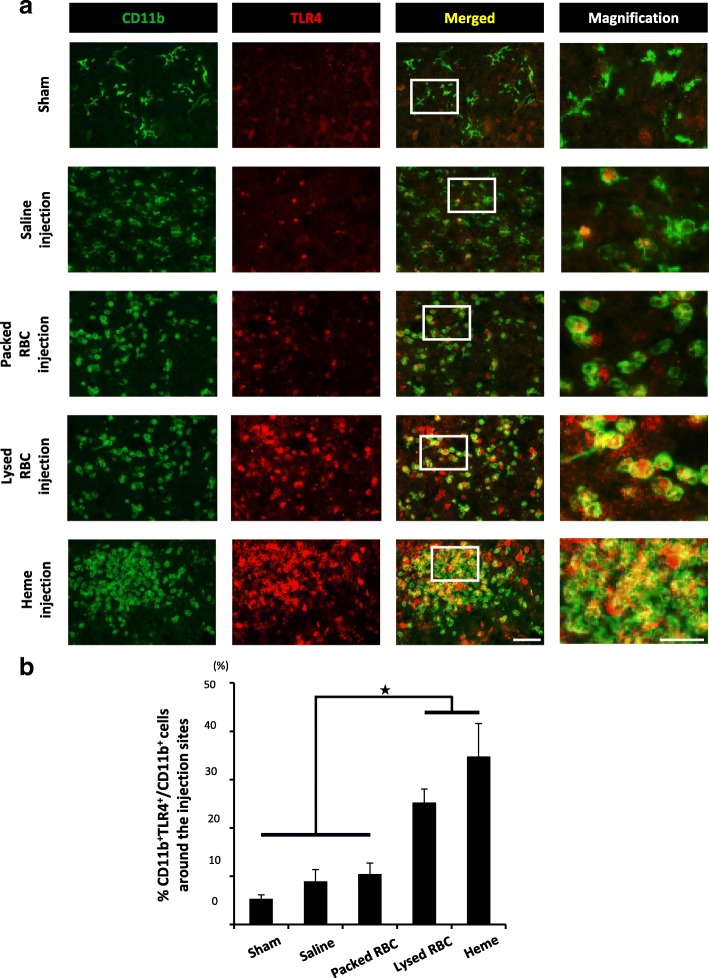
Heme induces the expression of TLR4 in CD11b-positive cells in the spinal cords. **a** At 1 day after the injections of saline, packed RBCs, lysed RBCs, or heme solution into the naïve spinal cord, sagittal sections were prepared. IHC staining around the injection sites showed the association between the expression of CD11b and TLR4. The right images are magnifications of the boxed area in merged images. **b** Quantification of the ratio of CD11b and TLR4 double-positive cells to CD11b single-positive cells of **a** (*n* = 6 in each group). Both Lysed RBCs and heme solution significantly upregulated the expression of TLR4 in CD11b-positive cells. Scale bars, 100 μm (a) and 40 μm in magnification. *p < 0.05, Dunnett’s test in comparison to sham-injected (laminectomy only) spinal cord (**b**). Error bars indicate the SEM

Microglial cells are resident macrophages in the CNS and participate in both innate and adaptive immune responses, including the induction of neuroinflammation via the release of proinflammatory cytokines/chemokines through antigen presentation [29]. Considering that heme is one of the major damage-associated molecular pattern molecules (DAMPs) in RBCs and that microglial cells recognize several DAMPs as antigens with TLR4 [30], we focused on the TLR4 expression in the microglial cells of the spinal cord. Although the injection of saline or packed RBC solution to the naïve spinal cord did not have any effect on the TLR4 expression in CD11b-positive microglial cells, the ratio of both CD11b and TLR4 double-positive cells to CD11b single-positive cells was significantly increased by the injection of lysed RBC solution (Fig. 3a, b). In addition, the ratio was further increased when heme solution was injected, suggesting that heme activated microglial cells via the TLR4 axis around the intralesional bleeding area (Fig. 3b).

### Heme exacerbates the TNF expression in microglial cells via TLR4 axis

**Fig. 4:**
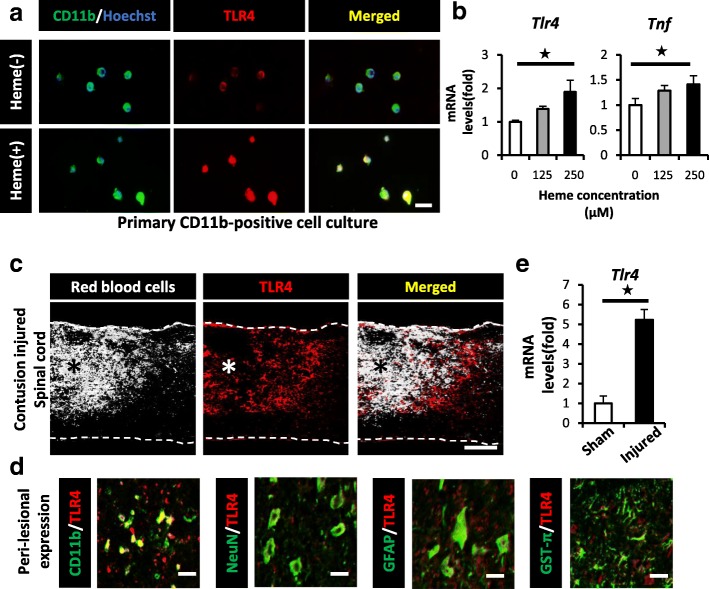
Heme exacerbates the TNF expression in CD11b-positive cells via the TLR4 pathway after SCI. **a** IHC showed heme-induced morphological changes and the expression of TLR4 (red) in primary CD11b-positive cells (green) at 30 min after heme stimulation (125 μM). **b** A qRT-PCR showed that the mRNA expression levels of TLR4 and TNF were both upregulated in a heme concentration-dependent manner (*n* = 4 in each group, triplicate). **c** IHC showed that intralesional bleeding (white) merged with TLR4-positive cells (red) in the peri-lesional area at 1 dpi. **d** IHC showed that CD11b-positive cells predominantly expressed TLR4 (red) around the lesion after SCI. CD11b, microglia; NeuN, neuron marker; GFAP, astrocyte marker; GST-π, oligodendrocyte marker (green). **e** The TLR4 mRNA expression in injured spinal cord at 1 dpi was significantly upregulated in comparison to sham spinal cord (*n* = 4 in each group). Scale bars, 20 μm (**a**, **d**), 500 μm (**c**). *p < 0.05, Wilcoxon rank-sum test (**e**) and Dunnett’s test in comparison to 0 μM heme-stimulation (**b**). Error bars indicate the SEM

We previously demonstrated that microglial activation was crucial for triggering the inflammatory responses and the subsequent pathology after SCI [31]. In particular, we reported that the tumor necrosis factor (TNF) expression in microglial cells was significantly associated with oligodendrocytic apoptosis and the functional prognosis of SCI [31–33]. We thus hypothesized that intralesional heme induced the expression of TNF in microglial cells and the subsequent demyelination after SCI. We isolated CD11b-positive microglial cells from the spinal cord tissue using magnetic beads and examined their in vitro response to heme stimulation. Consequently, we confirmed that heme stimulation resulted in the morphological transformation of the microglial cells and upregulated the expression of TLR4 (Fig. 4a). Furthermore, we found that heme stimulation significantly upregulated the expression levels of both TNF and TLR4 in microglial cells in a dose-dependent manner (Fig. 4b).

In accordance with our in vitro results, the TLR4 expression was predominantly observed in the microglial cells around the intralesional bleeding area of the injured spinal cord (Fig. 4c, d). In addition, the TLR4 expression was also significantly upregulated in the injured spinal cord in comparison to the sham-operated spinal cord (Fig. 4e). These results suggested that the TLR4/TNF axis was induced in the microglial cells around the lesion epicenter by intralesional heme.

### The administration of TXA reduced the toxic influence of heme and ameliorated the SCI pathology

To access the mechanism behind the decreased demyelination induced by the administration of TXA (Fig. 1e, f), we compared the heme content, the expression levels of TLR4 and TNF, and the number of apoptotic cells in the injured spinal cord among the three groups.

**Fig. 5:**
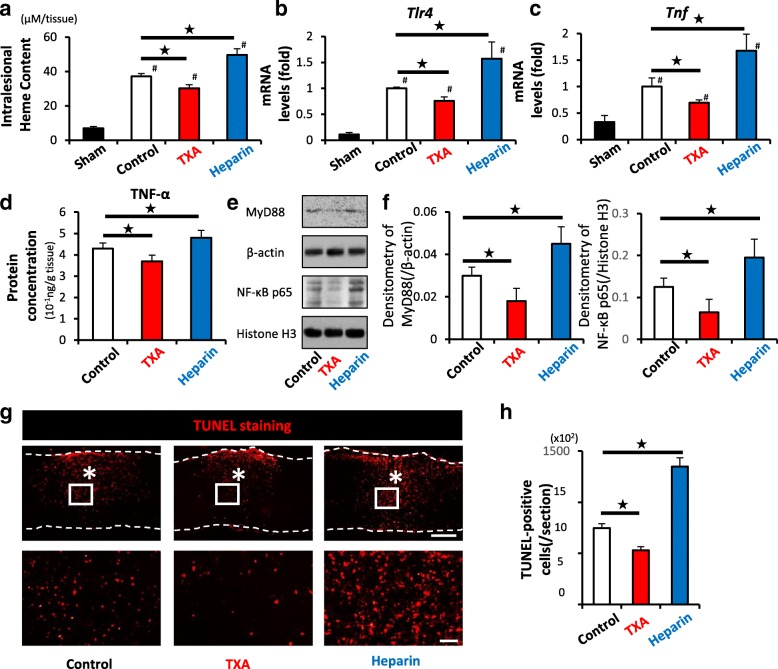
TXA alleviates the intralesional heme content, TLR4/TNF axis, and the number of apoptotic cells after SCI. **a** The intralesional heme concentration in injured spinal cords was increased in comparison to sham spinal cords at 1 dpi (#) (*n* = 6 in each group). The administration of TXA significantly reduced the heme content (black star). **b**, **c** A qRT-PCR showed that the administration of TXA significantly downregulated both the TLR4 and TNF mRNA expression in injured spinal cord at 1 dpi (*n* = 4 in each group). **d** Protein concentration of TNF-α of TXA-treated mice was significantly reduced compared to saline-treated mice at 3 dpi (*n* = 9 in each group). **e**, **f** Densitometric scanning of the immunoblots of MyD88, and NF-κB p65 (*n* = 9 per group). *p < 0.05, Dunnett’s test. The error bars indicate the SEM. **g** TUNEL staining showed the presence of TUNEL-positive apoptotic cells (red) around the lesion epicenter at 1 dpi. Bottom figures show higher magnification views corresponding to the boxed areas in the top figures. **h** Quantification of **e** demonstrating that the administration of TXA significantly reduced the number of TUNEL-positive apoptotic cells (*n* = 6 in each group). Scale bars, 500 μm (e left), and 50 μm (**e** right). *p < 0.05, Dunnett’s test (**a**–**d**, **f**, **h**) in comparison to control mice (black star) and sham-treated (laminectomy only) mice (#). Error bars indicate the SEM

Although the heme content in the injured spinal cords dramatically increased at 1 day after SCI in comparison to that in the naïve spinal cord, this increase in the intralesional heme content was significantly suppressed by the administration of TXA in comparison to the administration of saline (Fig. 5a). The expression of TLR4 and TNF and the number of TUNEL-positive apoptotic cells in the injured spinal cord were also significantly decreased by the administration of TXA compared with those in saline-treated mice (Fig. 5b–d, g, h). In contrast, heparin-treated mice showed a significant increases in heme content, the expression of TLR4 and TNF, and the number of apoptotic cells compared with those values in saline-treated mice (Fig. 5a–d, g, h). These results suggested that the intralesional heme content was associated with the expressions of TLR4 and TNF in vivo.

To confirm the effect of TXA on the Heme/MyD88/TNF axis after CNS bleeding as previously reported [30], we performed a Western blot analysis for MyD88 and NF-κB p65. As expected, the administration of TXA significantly reduced the molecules associated with this axis (Fig. 5e, f). These results suggested that TXA was able to improve the pathological prognosis by reducing heme-related neuroinflammation after SCI.

### The administration of TXA significantly improved the functional recovery after SCI

**Fig. 6:**
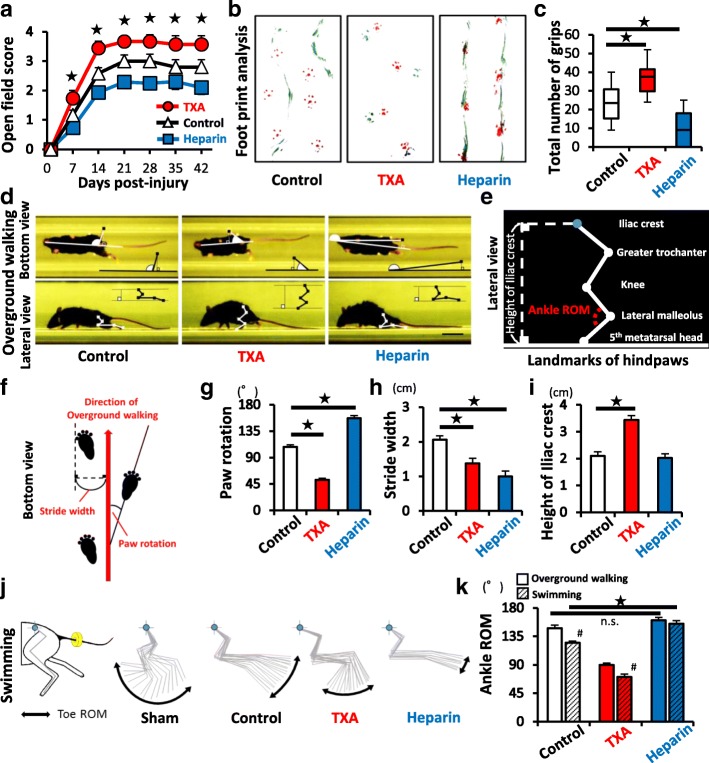
TXA improves functional recovery after SCI. **a** The open field score (BMS) was determined at each time point after SCI (TXA: *n* = 15, heparin: *n* = 14, control: *n* = 15). **b**, **c** The results of the footprint analyses (red, forepaws; green, hindpaws) and the grid walk test at 6 weeks after SCI (TXA: *n* = 15, heparin: *n* = 14, control: *n* = 15). **d** Bottom and lateral view in overground walking in MotoRater®. **e** A schematic illustration of the hindpaw landmarks (lateral view). **f** The definition of the direction of movement, paw rotation and stride width in overground walking (bottom view). **g**–**i** The results of MotoRater® measurement based on the definitions of **d**–**f**. **j** The kinetic parameters of MotoRater® measurement during swimming. Blue dot indicates the iliac crest. Yellow circle around the tail indicates a floating ring to increase buoyancy. **k** Quantification of ankle ROM in both overground walking and swimming. (TXA: *n* = 15, heparin: *n* = 14, control: *n* = 15). The swimming analysis showed greater sensitivity in detecting the improvement of the ROM than the overground walking analysis. Scale bars, 1 cm (**d**). *p < 0.05, two-way repeated-measures analysis of variance (ANOVA) with the Tukey-Kramer post-hoc test (**a**) and Dunnett’s test (**c**, **g**–**i**) in comparison to control mice. Wilcoxon rank-sum test between overground walking and swimming (#) and Dunnett’s test in comparison to control mice (black star) (**k**); *n*.*s*. not significant. Error bars indicate the SEM

For the clinical application of TXA in the treatment of SCI, we finally assessed the effect of TXA on the locomotor functional recovery. As a result, TXA-treated mice showed a better functional recovery in the open field motor score (Fig. 6a), foot print analysis (Fig. 6b), and grid walk test (Fig. 6c) in comparison to those of saline-treated mice. In this study, TXA-treated mice did not show behaviors indicative of spontaneous pain or mechanical allodynia (data not shown).

To clarify the functional differences among the three groups more precisely and objectively, we performed a novel quantitative and multifaceted assessment with a MotoRater® device [34] (Fig. 6d–k). The MotoRater® can automatically record the performance of several mice with a frame rate of 200 Hz by using a mobile high-speed camera. In overground walking, TXA-treated mice exhibited a smaller angle of paw rotation (Fig. 6g) and more parallel stride (Fig. 6h) in comparison to saline-treated mice when viewed from the bottom side. Furthermore, the height of the iliac crest of TXA-treated mice was maintained at a higher position in comparison to saline-treated control mice when viewed from the lateral side (Fig. 6i). In the supplemental analyses during swimming activities to eliminate the influence of gravity, TXA-treated mice exhibited significantly a wider range of ankle motion than in overground walking, whereas heparin-treated mice did not show a significant difference between overground walking and swimming (Fig. 6j, k; see Additional files 4, 5, 6, and 7). These objective results reinforced the notion that the administration of TXA was a feasible treatment for improving the functional recovery after SCI.

## Discussion

In this study, we suggested three major findings. First, contusion SCI brought about long-lasting RBC extravasation in the lesion, which correlated with the heme content released from lysed RBCs. Second, the intralesional heme was associated with the pathological processes of SCI, such as demyelination through the TLR4/TNF axis. Third, the administration of TXA after SCI successfully reduced the intralesional bleeding as well as the heme induction of the TLR4/TNF axis, which resulted in a better functional recovery after SCI.

In both the central nervous system (CNS) and other non-CNS tissues, discrete coagulation-fibrinolysis sequences are brought about when blood vessels are injured [35–37]. Not only after traumatic injuries but also under physiological conditions, the endothelial cells of blood vessels are constantly exposed to the minor injury by oxidative stress [38]. To repair the minor injured sites and maintain the patency/integrity of vessels, blood coagulation-fibrinolysis reactions are always appropriately regulated by several related factors. Among them, tissue plasminogen activator (t-PA) is mainly expressed in endothelial cells and constantly regulates these fibrinolysis reactions [39]. Notably, in contrast to the non-CNS tissues, t-PA is reported to be expressed in both the endothelial cells and neural cells in CNS tissues [40]. In fact, t-PA in the CNS is gaining a great deal more attention because of its involvement in various essential functions, including the development of the CNS, the plasticity of neural cells, the regulation of the blood-spinal cord barrier (BSCB) permeability, and proteolysis of the extracellular matrix [41–48]. In other words, the spinal cord has a distinct microenvironment with rich t-PA both inside and outside of the blood vessels in comparison to non-CNS tissues, leading to an excessive fibrinolysis reaction and prolonged intralesional bleeding after SCI. In previous studies, excessive and delayed bleeding after traumatic brain injury did not occur in t-PA-knocked out mice [6], suggesting that t-PA was primarily responsible for the long-lasting intralesional bleeding after SCI. Since TXA, an amino acid lysine analogue used in the present study, can bind with the lysine binding site of t-PA and inhibit the fibrinolysis reaction [13, 14, 49], the prolonged intralesional bleeding associated with t-PA is suggested to be subject to the suppressive effect of TXA on the intralesional hyperfibrinolysis reaction after SCI.

In the clinical setting, TXA is generally considered to enhance the hemostasis reaction through its anti-fibrinolytic effect. TXA is already used to reduce bleeding volume in surgery and after trauma [50, 51]. In this study, TXA reduced the intralesional bleeding volume and improved the locomotor function. Our studies are the first to demonstrate the therapeutic potential of TXA after SCI. In addition to the hemostatic effect, TXA is reported to reduce the mortality of hemorrhagic trauma patients [52, 53]. Whether this life-saving effect of TXA is simply a result of the anti-hemorrhagic effect or another effect has remained unclear. A large-scale clinical trial is in progress to elucidate the mechanism underlying the effects of TXA treatment. In this trial, prospectively registered traumatic brain injury patients are administered either TXA or placebo in a double-blind manner and the hemorrhage volume, functional outcome, mortality, thrombotic event rate, and other factors are evaluated [54, 55]. Although this clinical trial will provide insight into the therapeutic efficacy and risk of TXA in the management of traumatic brain injury, basic animal studies are necessary to understand the direct cause-effect mechanisms. In this study, we used both hemorrhagic and hemostatic murine SCI models, and proposed that the attenuation of heme cytotoxicity as well as the anti-hemorrhagic effect were the direct mechanisms through which the administration of TXA achieved better functional improvement after SCI.

In several hemolytic diseases, free heme is known to be a ligand to receptors expressed on various immune cells [56] and functions as major DAMPs, which can initiate the inflammatory cascade via TLR signaling [57]. For example, in intracranial hemorrhage, free heme induces microglial activation via TLR4 and the MyD88/TRIF signaling pathway, resulting in the increased expression of TNF [30, 58, 59]. In septic conditions, free heme synergistically upregulates the TNF expression in macrophages via the LPS axis, leading to further deterioration of the inflammatory reaction [60]. To counteract the heme-cytotoxicity, mammals are equipped with heme-scavenging enzymes, such as Hmox and Hpx. As reported by Lin et al. [61], Hmox-1-overexpressing rats exhibited less neural death and a better functional recovery after SCI than wild-type rats. Similarly, Han et al. [62] reported that Hpx^−/−^ mice showed a delayed recovery and exacerbated behavioral dysfunction after SCI while Hpx-overexpressing mice had an improved functional recovery. These reports suggested that heme and heme-related enzymes after SCI are associated with the SCI pathology via the TLR4/TNF axis in microglial cells and that modifying these enzymes may be a potential therapeutic option.

Thus far, TXA has never been applied in the treatment of SCI due to the following concerns. First, the administration of high-dose TXA increases the risk of seizure in a dose-dependent manner [63]. However, the TXA dosage in this study was approximately 81.4 mg/kg when converted to human dosage with interspecies allometric scaling [64], which seems to be reasonable considering the dosage currently applied in the clinical setting (100 mg/kg or 1 g) [54, 55, 65, 66]. In addition, TXA-treated mice never exhibited seizures in our experiments. Second, TXA is reported to increase the risk of thrombosis through its relatively coagulation-enhancing effect [50], and the administration of TXA to patients with hyperfibrinolytic conditions, such as DIC with the thrombotic phenotype, should be contraindicated [67]. However, unexpectedly, recent meta-analyses reported that vascular occlusive events were not significantly increased by the administration of TXA to traumatic brain injury patients [68, 69]. This discrepancy may come from the difference in the mechanism of hyperfibrinolysis. While the secondary hyperfibrinolysis after DIC is induced by consumption coagulopathy, the primary hyperfibrinolysis after CNS hemorrhage is suggested to be induced by an increase of t-PA [35]. Thus, in SCI, the administration of TXA can be expected to successfully inhibit the t-PA action, suppress primary hyperfibrinolysis, and exhibit a hemostatic effect without significant adverse effects. Third, the ‘TXA paradox’ is a matter of concern. TXA can sometimes accidentally turn on an excessive fibrinolytic reaction, resulting in a paradoxical increase of the bleeding volume [6, 70]. Indeed, the delayed administration of TXA after hemorrhagic trauma has been reported to increase the risk of death due to bleeding, whereas the acute administration of TXA is reported to reduce the risk [52, 53]. Regarding the mechanisms of the ‘TXA paradox’ after CNS hemorrhage, Medcalf et al. focused on the relationship between t-PA and urokinase plasminogen activator (u-PA) [70]. They showed that u-PA levels began to rise after t-PA levels had subsided and peaked at a later phase of injury [70]. Although TXA can block t-PA-mediated fibrinolysis, u-PA-mediated fibrinolysis is reversely promoted by TXA [70, 71]. Thus, to achieve a favorable outcome with the administration of TXA after SCI, without above concerning side effects, prudent selection regarding the timing of administration, as well as prospective clinical trials will be necessary.

## Conclusions

The administration of TXA reduced intralesional heme cytotoxicity and improved the functional prognosis after SCI. Although intralesional heme exacerbates the SCI pathology by activating microglial cells around the lesion epicenter, TXA attenuated the local hemolysis and reduced the heme induction of the TLR4/ TNF axis. We propose the administration of TXA as a promising treatment for patients with SCI.

## Additional files

Additional file 1: **Figure S1.** The schedule of the administration of saline, TXA, and heparin. (PPTX 33 kb)

Additional file 2: **Figure S2.** The accuracy of the actual impact force in TXA-, heparin-, and saline-treated SCI groups is shown (*n* = 14–15 mice per group, *p* < 0.05). (PPTX 54 kb)

Additional file 3: **Table S1.** The primer sequences used for qRT-PCR in the present study. Accession numbers are for the NCBI Gene database: https://www.ncbi.nlm.nih.gov/gene (XLSX 10 kb)

Additional file 4: **Movie S1.** Swimming analyses could sensitively detect the spared function after SCI (Movie S1: Naïve mice (MP4 513 kb)

Additional file 5: **Movie S2.** Saline-treated control mice (MP4 487 kb)

Additional file 6: **Movie S3.** TXA-treated mice (MP4 484 kb)

Additional file 7: **Movie S4.** Heparin-treated mice). Representative movies captured during the swimming analyses (Fig. 5j) are presented (MP4 510 kb)

## Data Availability

All data generated or analyzed during this study are included in this published article and its supplementary information files.

## Abbreviations

b.w: Body weight
BMS: Basso Mouse Score
BSCB: Blood-spinal cord barrier
CNS: Central nervous system
DAMPs: Damage-associated molecular patterns
DIC: Disseminated intravascular coagulation
DMEM: Dulbecco’s modified Eagle’s medium
dpi: Days post-injury
FBS: Fetal bovine serum
FDP: Fibrin/fibrinogen degradation product
GAPDH: Glyceraldehydes-3-phosphate dehydrogenase
GFAP: Glial fibrillary acidic protein
Hmox1: Heme oxygenase 1
Hpx: Hemopexin
i.p.: Intraperitoneal injection
LFB: Luxol fast blue
LPS: Lipopolysaccharide
PBS: Phosphate-buffered saline
PFA: Paraformaldehyde
qRT-PCR: Quantitative reverse transcription polymerase chain reaction
RBC: Red blood cell
ROS: Reactive oxygen species
s.c.: Subcutaneous injection
SCI: Spinal cord injury
TLR4: Toll-like receptor 4
TNF: Tumor necrosis factor
t-PA: Tissue-plasminogen activator
TUNEL: Terminal deoxynucleotidyl transferase-mediated dUTP nick-end labeling
TXA: Tranexamic acid
u-PA: Urokinase plasminogen activator
