# Supplementary material for: Tranexamic acid reduces heme cytotoxicity via the TLR4/TNF axis and ameliorates functional recovery after spinal cord injury
Source: J Neuroinflammation. 2019 Jul 29;16:160. doi: 10.1186/s12974-019-1536-y (PMC6661785; doi:10.1186/s12974-019-1536-y)
Supplement: Supplementary file 1 — Figure S1. The schedule of the administration of saline, TXA, and heparin. (PPTX 33 kb) [file 12974_2019_1536_MOESM1_ESM.pptx]

## Slide 1
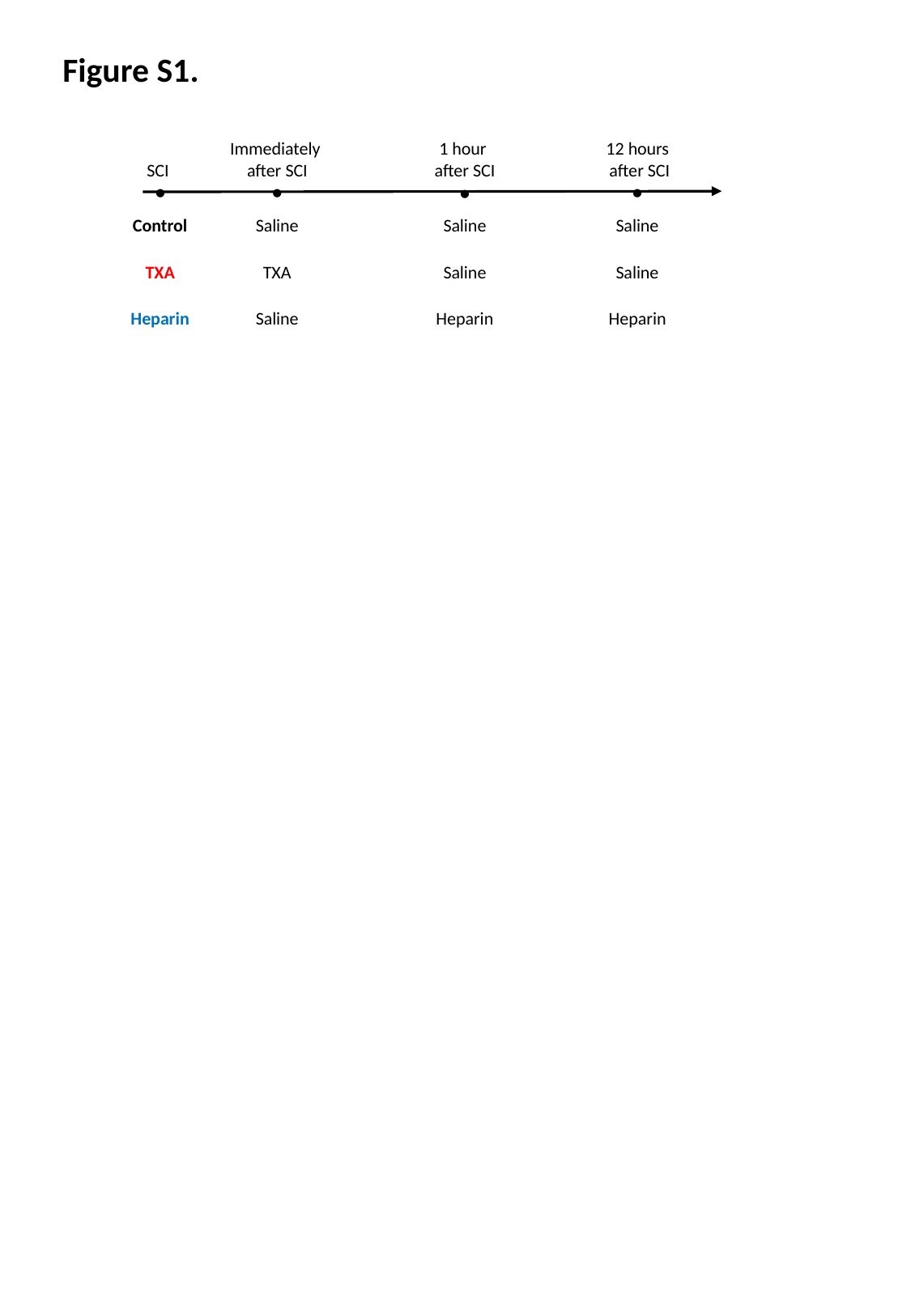

Figure S1.
Immediately
after SCI
1 hour
after SCI
12 hours
 after SCI
SCI
●
●
●
●
Control
Saline
Saline
Saline
TXA
TXA
Saline
Saline
Heparin
Saline
Heparin
Heparin
