# Supplementary figures and images for: Tranexamic acid reduces heme cytotoxicity via the TLR4/TNF axis and ameliorates functional recovery after spinal cord injury
Source: J Neuroinflammation. 2019 Jul 29;16:160. doi: 10.1186/s12974-019-1536-y (PMC6661785; doi:10.1186/s12974-019-1536-y)

## Slide 1
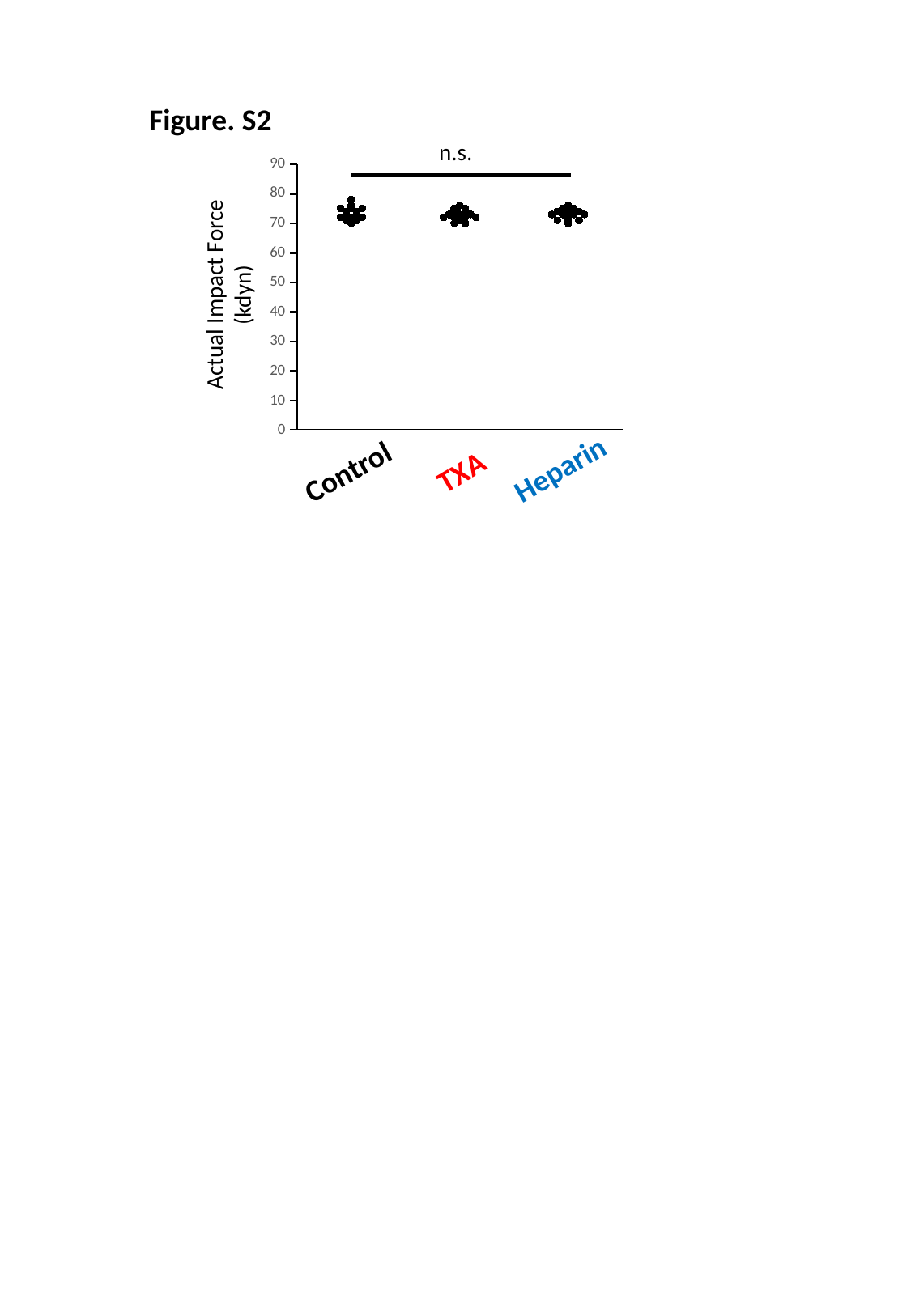

Figure. S2
n.s.
### Chart
| Category | |
|---|---|Actual Impact Force
(kdyn)
Heparin
TXA
Control

Supplement: Supplementary file 2 — Figure S2. The accuracy of the actual impact force in TXA-, heparin-, and saline-treated SCI groups is shown (n = 14–15 mice per group, p < 0.05). (PPTX 54 kb) [file 12974_2019_1536_MOESM2_ESM.pptx]
